# “Textbook outcome(s)” in colorectal surgery: a systematic review and meta-analysis

**DOI:** 10.1007/s11845-024-03747-w

**Published:** 2024-07-10

**Authors:** Benjamin M. Mac Curtain, Wanyang Qian, Aaron O’Mahony, Avinash Deshwal, Reuben D. Mac Curtain, Hugo C. Temperley, Niall O. Sullivan, Zi Qin Ng

**Affiliations:** 1 Department of Urology, St Vincent’s University Hospital, Dublin, Ireland; 2 Dept of General Surgery, St John of God Midland Hospital, Midland, WA Australia; 3 Department of Surgery, University Hospital Limerick, Limerick, Ireland; 4 Department of Surgery, Fiona Stanley Hospital, Perth, WA Australia; 5 School of Medicine, University College Dublin, Dublin, Ireland; 6 Department of Surgery, St James’ University Hospital, Dublin, Ireland; 7 Department of General Surgery, Royal Perth Hospital, Perth, WA Australia

**Keywords:** Colon and rectal surgery, Colorectal cancer, Colorectal surgery, Survival, Textbook outcome(s)

## Abstract

**Background:**

Textbook outcome (TO) is a composite measure used in surgery to evaluate post operative outcomes. No review has synthesised the evidence in relation to TO regarding the elements surgeons are utilising to inform their TO composite measure and the rates of TO achieved.

**Methods:**

Our systematic review and meta analysis was conducted in line with the Preferred Reporting Items for Systematic reviews and Meta-Analyses (PRISMA) recommendations. PubMed, EMBASE, and Cochrane central registry of controlled trials were searched up to 8th November 2023. Pooled proportions of TO, clinical factors considered and risk factors in relation to TO are reported.

**Results:**

Fifteen studies with 301,502 patients were included in our systematic review while fourteen studies comprising of 247,843 patients were included in our meta-analysis. Pooled rates of TO achieved were 55% with a 95% confidence interval (95% CI) of 54–55%. When stratified by elective versus mixed case load, rates were 56% (95% CI 49–62) and 54% (95% CI 50–58), respectively. Studies reported differing definitions of TO. Reported predictors of achieving TO include age, left sided surgery and elective nature.

**Conclusions:**

TO is achieved, on average in 55% of reported cases and it may predict short and long term post operative patient outcomes. This study did not detect a difference in rates between elective versus mixed case load TO proportions. There is no standardised definition in use of TO. Standardisation of the composite is likely required to enable meaning comparison using TO in the future and a Delphi consensus is warranted.

**Supplementary Information:**

The online version contains supplementary material available at 10.1007/s11845-024-03747-w.

## Introduction

Conventional quality measurement has relied on assessing individual outcome indicators such as post operative complications, mortality and length of hospital stay (LOS). Composite outcome measures, may be more meaningful and clinically relevant, combining the multi-dimensional aspect of the complex surgical process into a single indicator. This also allows for ease of comparison across institutions when assessing quality of care [1].

“Textbook outcome” (TO) accounts, not only for the postoperative outcomes related to surgical morbidity but also the ideal oncologic result [2]. TO in colon and rectal cancer surgery was first proposed in 2013, with 6 desired outcomes; hospital survival, radical resection, no major complications, no reintervention, no unplanned stoma and no prolonged LOS or readmission. A ‘textbook’ hospital stay was set at the 75th percentile of the population / a hospital stay of 14 days or less. When all 6 desired health outcomes were realised, a TO was achieved [3].

Multiple studies have shown that when a TO is met it is associated with improved long-term survival [4–6]. TO has been studied across oesophagogastric, pancreatic, liver, and transplant surgery. In a systematic review conducted by Carbonell-Morote et al. it was found that 58.3% of patients who achieved TO following oncological gastric surgery had a significant increase in long term survival [7]. One study suggested a 4%—12% improvement in overall survival (OS) for every 10% increase in the adjusted hospital TO [8]. From this, it may be inferred that TO is an indicator for short term quality of care and a predictor of long-term outcomes.

To ensure effectiveness, TO must be replicable with readily adjustable parameters. Controversy arises when there’s disagreement on the definition. For instance, in colorectal cancer, opting for a stoma to prevent an anastomotic leak can be a justifiable decision, though it may not align with the concept of a TO [9]. Non-modifiable variables such as patient anatomy and cancer biology can have a significant influence on TO and long-term survival. Auer et al. report the overall rate of TO achievement for colon cancer is approximately 67% versus rectal cancer at less than 34%, even with similar patient demographic, surgeons, and hospital processes [2].

TO assumes a “textbook” patient and this does not encompass the variety of patients encountered in surgical oncology. However, despite its limitations TO is a useful quality assessment tool and has been shown to be a significant prognostic indicator in survival. This systematic review and meta-analysis aims to report the pooled proportions of TO achieved in colon and rectal surgery, as well as detailing what studies have utilised to comprise TO. Predictors of TO and any patient outcomes will also be reported.

## Methods

### Registration and search strategy

Our search was conducted in line with the most recent Preferred Reporting Items for Systematic reviews and Meta-Analyses (PRISMA) recommendations [10]. The study was registered on PROSPERO under the reference CRD42023489352. A search of PubMed, EMBASE and Cochrane Central Register of Controlled Trials was conducted utilising the search algorithms provided below, up to the 8th November 2023:

- "textbook outcome*" and ("colo*" OR "rectal" OR "anal") – PubMed and EMBASE
- *(textbook outcome) and (colorectal or rectal or anal or colon)- Cochrane*

**Fig. 1:**
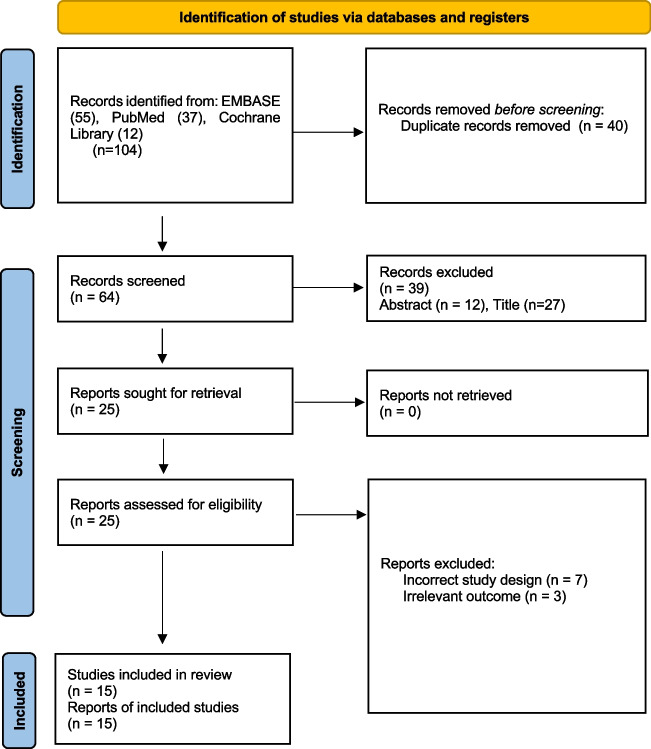
PRISMA Statement for textbook outcome in colorectal surgery.

The complete breakdown of analysed studies can be viewed in the PRISMA diagram in Fig. 1. The bibliographies of included publications were also searched for any relevant studies.

Inclusion criteria.

- English language or translation available.
- Studies detailing the rates of TO and elements compiled to define TO.
- Studies detailing TO in relation to colorectal surgery.
- Adult patients > 18 years old.
- Retrospective or prospective clinical studies.

Exclusion criteria.

- Non fulfilment of the inclusion criteria.
- Studies with metastatic resection at time of colorectal surgery.
- Patients with synchronous or metachronous cancer.
- Case series defined as <  = 10 patients, case reports, or any type of review.

## Identification of studies and outcomes of interest

Studies that satisfied the inclusion and exclusion criteria were included. The following PICO elements were used as the basis for selecting studies [11]:

- *Population*: Patients undergoing colorectal surgery in whom achievement of TO was recorded.
- *Intervention*: Achievement of TO.
- *Comparison*: Non achievement of TO.
- *Outcome*: Rates of TO, elements comprising TO and outcomes / predictors in relation to TO.

Studies were independently reviewed by two separate authors (BMC, WQ) using Rayyan [12]. If there was any disagreement between authors a third author (AD) was used to mediate the discussion and consensus was reached.

Our primary outcome of interest was the rate of TO achieved post colorectal surgery.

Secondary outcomes of interest were the elements used to define TO, predictors of achieving TO and the patient outcomes when TO was achieved.

## Data extraction

Study demographics and TO variables of concern were transcribed using Google Sheets (Mountain View, California, United States). Four independent authors (WQ, AD, BMC, RMC) were involved in the data extraction.

## Study selection

Prospective and retrospective studies were included in this systematic review and meta-analysis. No randomised trials have been completed on the topic to the best of the author’s knowledge.

Both the rates and definition of TO were used as the primary criterion for inclusion. If studies reported risk factors / predictors or patient outcomes in relation to TO, this data was also reported and was meta-analysed if sufficiently homogeneous. If a study did not report elective or non-elective status they were excluded from this sub-analysis.

## Risk of *Bias* assessment

Assessment of potential biases for non-randomised studies was assessed using a modified Newcastle–Ottawa scale risk of bias tool [13], with the results tabulated in Table 1. This assessment tool grades each study as being ‘satisfactory’ or ‘unsatisfactory’ across various categories. We assigned stars to evaluate study quality: 7 stars—“very good”, 5–6 stars “good”, 3–4 stars “satisfactory” and 0–2 stars “unsatisfactory”. The critical appraisal was completed by two reviewers independently (HT and WQ), where once again a third reviewer (BMC) was asked to arbitrate in cases of discrepancies in opinion.

**Table 1:**
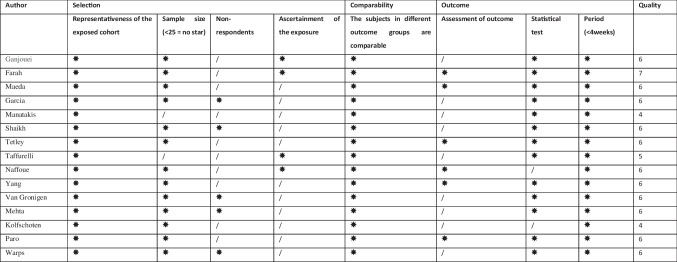
Risk of bias assessment

## Statistical analysis

We performed a proportional meta-analysis as part of this review [14]. Statistical analysis was run using Stata 17 (StataCorp. 2021. *Stata Statistical Software: Release 17*. College Station, TX: StataCorp LLC). Proportions were pooled using the “metaprop” function within Stata [15]. 95% confidence intervals were employed and *p* <  = 0.05 was considered statistically significant. Heterogeny was reported using I^2^ [15]. It has been put forward that I^2^ values of 25%, 50%, 75% can be used to assess the degree of heterogeneity [16]. We considered there to be a notable degree of heterogeny if I^2^ was greater than 50%. A random effects model was used due to evidence of significant statistical heterogeneity as well evidence of study design heterogeneity [17].

Funnel plots were not generated as previously recommended for proportional meta-analysis [18]. Qualitative bias assessment was also conducted as proposed by Barker et al., as this is a proportional meta-analysis [14].

## Results

### Primary outcome

Fifteen studies with 301,502 total participants were included in our systematic review and analysis as shown in supplemental Table 1 [3–5, 19–30]. One study was excluded from our meta-analysis due to being a propensity matching study [20]. All studies were conducted retrospectively and published in the range 2013–2023. Shaikh et al. and Mehta et al. only included elderly patients aged 65 years or older [22, 28]. Four studies reported only including elective cases [20, 24, 28, 30]. Study demographics and inclusion criteria are included in Tables S1 and S2.

### Pooled proportion of TO

**Fig. 2:**
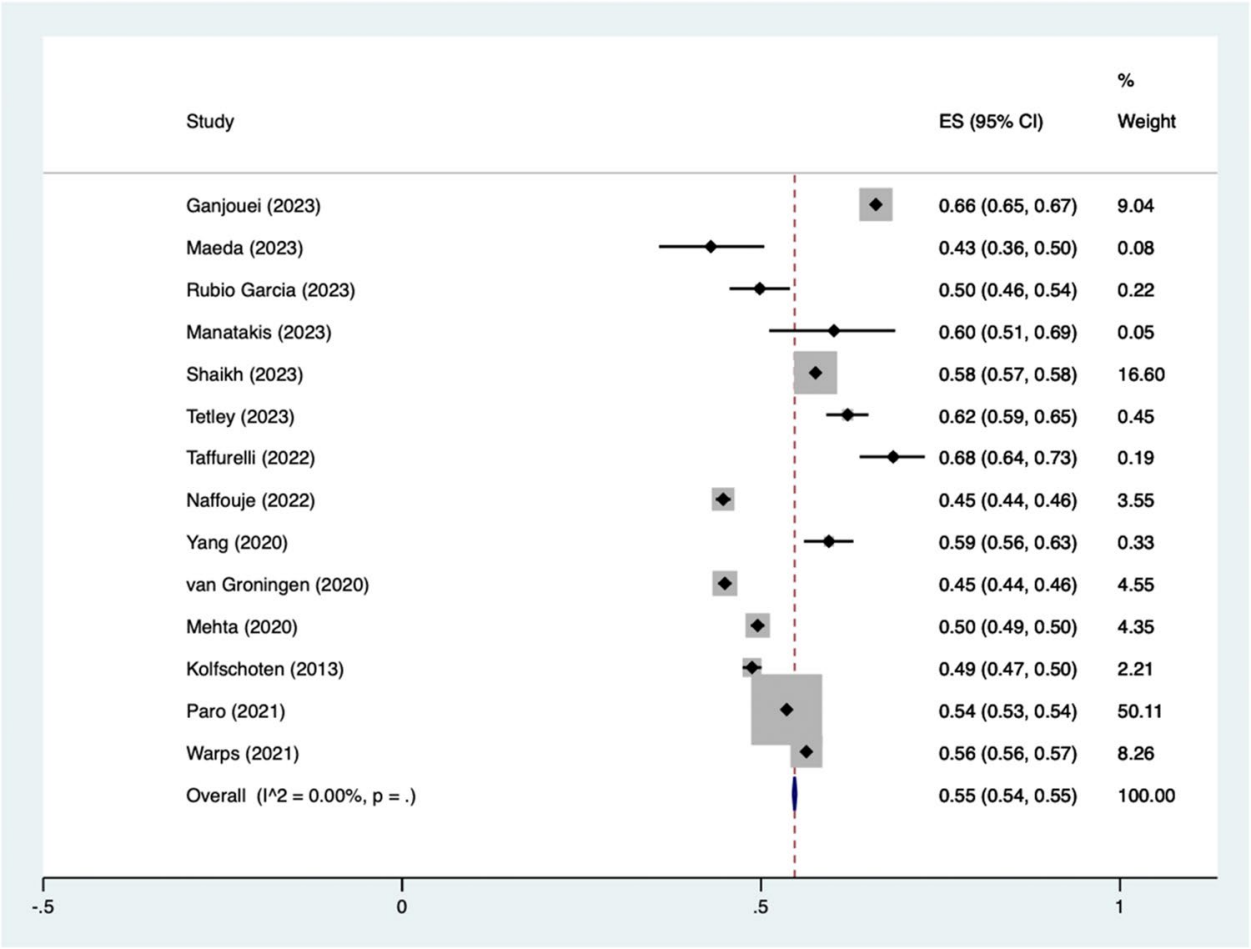
Pooled proportions of patients achieving a textbook outcome

**Fig. 3:**
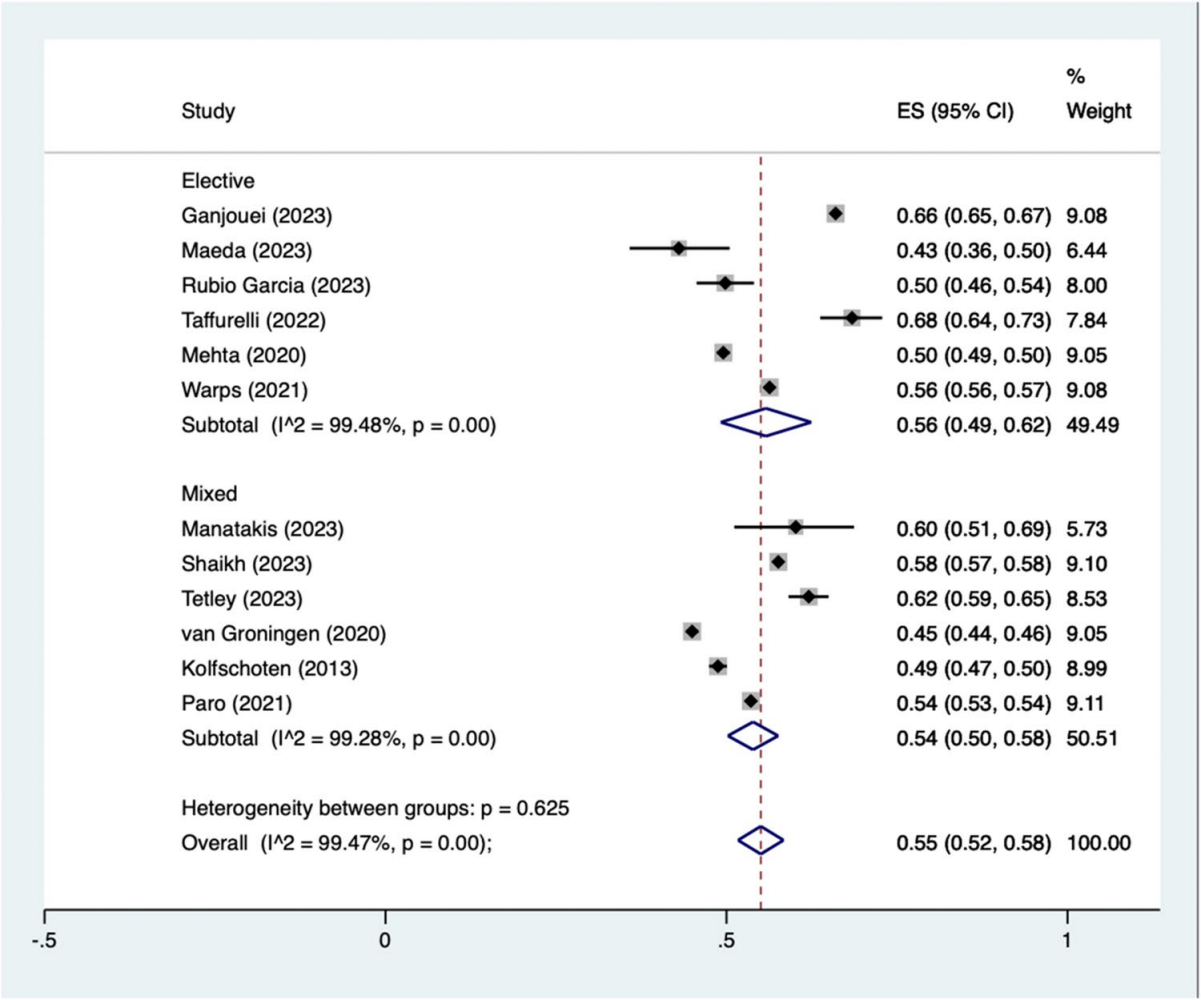
Pooled proportions of patients achieving a textbook outcome stratified by elective status

Fourteen studies were included in the analysis ( n = 247,843) as seen in Fig. 2. The pooled rate of patients being classified as achieving a TO was 55% (95% CI 54–55%). I^2^ was calculated as 0. One study comprised over half of the weighted percentage in our meta-analysis [29]. Studies were also stratified based on elective or mixed elective and emergency case load, as seen in Fig. 3 and Table S3. TO rates in elective surgery were 56% (95% CI 49–62) and 54% (95% CI 50–58) in mixed / non - elective. Individual rates of TO can be seen in Table S3.

### Secondary outcomes

#### Elements / outcomes used to comprise TO

Certain elements are used commonly by studies to comprise of TO as displayed in Table S3. Table S4 outlines peri and post operatives outcomes. Broad categories include standard post-operative outcomes: mortality, length of stay and readmission rates. Seven studies reported on 30-day mortality [4, 5, 19, 20, 23, 27, 30], whilst four studies reported on 90-day mortality [22, 24, 25, 29]. Kolfschoten et al. reported on hospital survival regardless of LOS [3]. Many studies reported prolonged LOS as an exclusion for TO. Ganjouei et al. defined prolonged LOS as longer than 5 days [19], whilst Rubio Garcia et al. and Kolfschoten et al. defined prolonged LOS as longer than 14 days [5, 3]. Others such as Naffouje et al. defined a suitable LOS as less than the 75th centile [25]. Readmissions were similarly reported within 30-days by four studies [5, 19, 20, 25] whilst one study reported on 90-day readmission rates [22].

Post-operative complications precluded achievement of TO in the majority of studies. Taffurelli et al. and Tetley et al. defined significant complications as Clavien Dindo grade greater than III and IV respectively [23, 24]. Another aspect of the TO is the achievement of sufficient oncological or radical resection of the tumour, known as R0 resection. This was included in the TO definition for eight studies [3–5, 21, 25–27, 30]. Three studies reported a lymph node yield of greater than or equal to twelve as a requirement for TO [5, 25, 26]. The formation of a stoma was also included in the TO definition – Maeda et al. excluded all cases with a stoma formation from TO definition, whilst Manatakis et al. excluded unplanned stomas from achieving TO [4, 21]. Stoma formation was reported within the range 2.3% to 33% in our included studies (4, 5, 3, 21, 26, 30).

#### Predictors of TO

Due to the heterogeneous aims of studies included in our analysis, many differing factors from a biopsychosocial model were found to be predictive in the achievement of a TO. As in Table S5 surgical factors such as approach was reported to be predictive of TO (TO after robotic colectomy (77%), lap colectomy (68%), open colectomy (39%), *p* < 0.001) [19]. Cancer factors such as staging and classification was also stated to be predictive of TO (T3 and T4 classification (OR 2.50, 95% CI 4.59–1.36, and OR 2.55, 95% CI 5.21–1.24 respectively) [5]. Patient factors such as age and gender were also found to be predictive of TO (68.5 average age versus 73.9 average age in TO versus non TO, *p* = 0.005) [4] (female gender AOR 1.599 95% CI 1.499–1.706 *p* < 0.001) [30]. Environmental factors were also significant in predicting TO; Taffurelli et al. found being a dependent in the living situation (*p* = 0.041) was a risk factor preventing patients from achieving TO on univariate analysis [24], whilst Shaikh et al. reported on multivariate analysis that patients residing in high Environmental Quality Index areas were likely to achieve TO (OR 0.94 95% CI 0.89–0.99 *p *= 0.02) [22].

Conversely, TO was also used to predict other outcomes, as Maeda et al. reported TO to be predictive of overall survival and relapse free survival compared to non-TO (OS, 77.8% vs. 60.8%, *P* < 0.01; RFS, 69.6% vs. 50.8%, *P *= 0.01) [21].

#### Risk of *Bias*

Risk of bias is presented in Table 1 using our modified Newcastle–Ottawa scale as described in the methods section. Two studies received a 4 [3, 4]. One received a 5 [24]. Eleven studies received a score of 6, while one study received a 7 [20].

## Discussion

This systematic review and meta-analysis examined rates of TO, elements incorporated into the TO composite measure, and the risk factors associated with TO achievement.

We report a pooled proportion of TO achievement of 55% (95% CI 54–55%). This represents 55% of patients achieving a TO as defined within their publications. TO rates in elective surgery were calculated as 56%, and 54% for mixed elective / urgent / emergency cases, the difference in rate was not statistically significant. The definitions of TO varied throughout each study, with all reporting on some combination of post operative complications, LOS, readmission rates, mortality. Composite measures have been shown as superior to singular metrics in regards to evaluating patient long term survival [31, 32]. Besides being a short term post operative composite measure, TO has been shown to correlate to increased patient 5 year survival post colon cancer surgery [26].

Studies have described their own definition of TO, including patients experiencing no complications or prolonged LOS amongst other self-defined metrics. At this point the need for a standardisation of the composite measure cannot be understated. It is difficult to meaningfully compare studies reporting TO, if they report differing definitions of the measure. Deciding upon set metrics for inclusion such as LOS may be difficult. Taking the example of LOS, if a patient was discharged on post operative day 6 instead of day 5 to receive stoma education that may not necessarily reflect a negative outcome, but rather a positive one [33]. Granted fashioning of a stoma may exclude one from achieving TO, however this example merely serves to illustrate a potential shortcoming of using TO without clinical context. Another issue which may warrant further research is the effect of neoadjuvant treatment on TO. Consideration should be given to having it incorporated into the measure as it may be necessary to control for differing surgical difficulty and complication rates post neoadjuvant treatment and surgery [34]. Additionally, the inclusion of surgical histopathologic results may be warranted which has been shown to change from the initial pathology report and may potentially alter surgical post operative outcomes [35]. There is a lack of multidisciplinary inclusion in the current TO model. The authors believe a Delphi consensus regarding variables used to comprise TO, is warranted.

The use of TO has a role to play in the evaluation of patient outcomes post colorectal surgery, however it is prudent to not use it at this time to evaluate surgical performance due to the lack of clinical context, rather the authors view TO as a quality improvement measure or possibly as a predictor for patient outcomes post-surgery. Robotic and laparoscopic approaches, elective cases, and left sided surgery were all found to be predictive of achieving TO [4, 5, 23, 36]. Within our analysis the rates of TO were similar in elective colorectal surgery at 56%,compared to 54% in mixed cases, with no statistically significant difference. As well as this, patients undergoing rectal surgery were more likely to achieve TO compared to others [29]. The use of TO to stratify patients in most need of being enrolled in enhanced recovery after surgery programmes (ERAS) is also another potential application. Additionally, frailty assessment has been shown as predictive of TO achievement further illustrating the use of TO in the prediction of surgical outcomes [24].

There are a number of limitations to this review including the inherent limitations of the included studies. Studies were retrospective which introduces additional bias potential [37]. The heterogenous nature of the included studies not fully stratifying based on emergency / urgent status may alter the generalisability of our results. It has been previously shown that protocols can be put in place to better patient recovery in emergency cases, however this may be less effective than in an elective setting [38], the same is plausible in the case of TO. Additionally, TO are compiled using differing surgical approaches, which has been shown to lead to differing post operative patient outcomes [39–41]. There are also limitations of the statistics model employed [17]. All studies received at least a satisfactory grading in the risk of bias assessment with the majority receiving considerably higher.

## Supplementary Information

Below is the link to the electronic supplementary material.

Supplementary file1 (DOCX 42.4 KB)
