# Supplementary material for: “Textbook outcome(s)” in colorectal surgery: a systematic review and meta-analysis
Source: Ir J Med Sci. 2024 Jul 10;193(5):2187–94. doi: 10.1007/s11845-024-03747-w (PMC11450112; doi:10.1007/s11845-024-03747-w)
Supplement: Supplementary file 1 — Supplementary file1 (DOCX 42.4 KB) [file 11845_2024_3747_MOESM1_ESM.docx]

Table S1 Study demographic details and inclusion / exclusion criteria

| Author | Title | Year | Journal | Country | Study type | Number of patients | Inclusion Criteria | Exclusion Criteria |
| --- | --- | --- | --- | --- | --- | --- | --- | --- |
| Ganjouei | A Novel Machine Learning Approach to Predict Textbook Outcome in Colectomy. | 2023 | Diseases of the Colon and Rectum | USA | Retrospective | 20498 | >18 years old with of diagnosis non metastatic colon cancer who underwent elective colectomy from 2014-2020, American College of Surgeons (ACS) National Surgical Quality Improvement Program (NSQIP) database | No postoperative surgical complications, no prolonged length of hospital stay, no readmission 90 days after discharge, no mortality 90 days after surgery |
| Farah | Perioperative outcomes of robotic and laparoscopic surgery for colorectal cancer: a propensity score-matched analysis. | 2023 | World Journal of Surgical Oncology | USA | Retrospective | 53209 | ACS NSQIP database 2015-2020, current procedural terminology codes for colorectal procedures, including elective robotic/laparoscopic resections with anastomosis for CRC | Disseminated cancer, ascites, preoperative sepsis, ASA 5, ventilator dependence, concurrent major procedures eg. hepatectomy/pancreatectomy |
| Maeda | Textbook outcome contributes to long-term prognosis in elderly colorectal cancer patients. | 2023 | Langenbecks Archives of Surgery | Japan | Retrospective | 186 | Consecutive patients underwent curative surgery at a single centre. >75 year old, for CRC, pathological confirmed adenocarcinoma, stage I-III, curative surgery. | Pathological stage IV, missing data on clinicopathological characteristics |
| Rubio Garcia | Textbook outcome in colon carcinoma: implications for overall survival and disease-free survival. | 2023 | Langenbecks Archives of Surgery | Spain | Retrospective | 564 | Elective CRC resection with confirmed histological diagnosis | <18, emergency surgery, resection of rectum, resection for any pathology other than cancer, could not assess TO, Follow up <2 years |
| Manatakis | Achieving a Textbook Outcome in Colon Cancer Surgery Is Associated with Improved Long-Term Survival. | 2023 | Current Oncology | Greece | Retrospective | 128 | >18 years old, CRC patients treated in single institution. Non metastatic colon adenocarcinoma stage I-III, underwent colectomy with curative intent following complete mesocolic excision principles. | Recurrent and metastatic cancers, palliative/non-CME surgery. Rectal cancers and other histological types other than adenocarcinoma excluded. |
| Shaikh | Association between the environmental quality index and textbook outcomes among Medicare beneficiaries undergoing surgery for colorectal cancer (CRC). | 2023 | Journal of Surgical Oncology | USA | Retrospective | 40939 | >65 year old diagnosed with CRC selected from SEER database using ICD 9 and ICD 10 codes | Different primary cancer diagnosis, diagnosed solely through autopsy/death certificate, missing information on race/surgery/clinicopathological stage, date of death or country-level EQI status |
| Tetley | Association of Insurance Type With Colorectal Surgery Outcomes and Costs at a Safety-Net Hospital: A Retrospective Observational Study. | 2023 | Annals of Surgery Open: Perspectives on Surgical History, Education, and Clinical Approaches | USA | Retrospective | 1078 | All patients undergoing colorectal procedures present in the 2013-2019 NSQIP at a single facility | Perineal and trans-sacral only procedures, missing or inaccurate cost variables, and “Other” insurance type. |
| Taffurelli | Frailty assessment can predict textbook outcomes in senior adults after minimally invasive colorectal cancer surgery. | 2022 | European journal of surgical oncology | Italy | Retrospective | 421 | All consecutive patients ≥ 70 years of age who underwent elective surgery for colorectal cancer with curative intent between January 2017 and November 2021 | Patients who underwent either diagnostic laparoscopic procedures, palliative procedures or transanal local excisions |
| Naffouje | Assessment of Textbook Oncologic Outcomes Following Proctectomy for Rectal Cancer. | 2022 | Journal of gastrointestinal surgery | USA | Retrospective | 8,869 | All patients presenting with localised rectal adenocarcinoma (nonmetastatic stage T3/T4 N0 or T-any N+) who underwent proctectomy (abdonioperineal resection, left anterior resection, or pelvic exenteration) between 2010-2017 and only received single-agent neoadjuvant chemoradiation followed by proctectomy within 5-12 weeks of radiotherapy conclusion | Patients who received alternative doses/ fractions/ radiation to areas other than the pelvis/ rectum. Patients with different histologies, metastatic disease, multiple malignancies, those who underwent procedures other abdominal proctectomy and receipt of multi-agent neoadjuvant chemotherapy. Patients with missing information on surgical approach. Patients with missing pathologic data on resection margin status, postoperative LOS, receipt of systemic chemotherapy, readmission rates, and 30- and 90- day mortality |
| Yang | The association between the composite quality measure "textbook outcome" and long term survival in operated colon cancer. | 2020 | Medicine | Taiwan | Retrospective | 804 | All patients who underwent colon cancer resection with curative intent (right hemicolectomy, left hemicolectomy, or sigmoidectomy) | Carcinoma in-situ, a previous history of cancer, age <18 years, chemotherapy as the initial treatment or missing data |
| van Groningen | Identifying best performing hospitals in colorectal cancer care; is it possible? | 2020 | European journal of surgical oncology | Netherlands | Retrospective | 11373 | All patients that underwent surgery for primary colorectal cancer in the Netherlands in 2015 | Transanal local excision of rectal cancer, multiple synchronous tumours, patients with missing values in the outcomes |
| Mehta | Comparing textbook outcomes among patients undergoing surgery for cancer at U. S. News & World Report ranked hospitals. | 2020 | Journal of Surgical Oncology | USA | Retrospective | 10984 | >65 years old, not benign or emergency surgery, medicare inpatient data with ICD-9 colorectal surgery procedure codes | <65 years old, benign disease, emergency surgery |
| Kolfschoten | Focusing on desired outcomes of care after colon cancer resections; hospital variations in 'textbook outcome'. | 2013 | European journal of surgical oncology | Netherlands | Retrospective review of prospective database | 5582 | 1 year period in 2010, DSCA database, Patient undergoing surgery for colon cancer | Recurrent or synchronous colorectal tumours, failed to register all patients in 2010, failed to register all outcome parameters for more than 15% of patients, failed to fill in the required case-mix factors for more than 15% of patients |
| Paro | Impact of Residential Racial Integration on Postoperative Outcomes Among Medicare Beneficiaries Undergoing Resection for Cancer. | 2021 | Annals of surgical oncology | USA | Retrospective | 125896 (111185 colorectal, 14711 rectal) | Patients who underwent resection for lung, oesophageal, colon, and rectal cancer, data extracted from 100% Medicare Inpatient Standard Analytic Files between 2013 and 2017 | Patients who were not enrolled in Medicare Parts A and B, as well as patients who were enrolled in a Health Maintenance Organization (HMO) in the month of the surgical episode were excluded |
| Warps | Textbook outcome after rectal cancer surgery as a composite measure for quality of care: A population-based study. | 2021 | European Journal of Surgical Oncology | Netherlands | Retrospective | 20521 | Elective surgery for first primary rectal carcinoma between 2012 and 2019 | Multiple tumours or local excision, missing data on textbook outcome |

Table S2 Study patient demographics

| Author | Age | Gender | BMI | ASA | Comorbidities | Cancer stage |
| --- | --- | --- | --- | --- | --- | --- |
| Ganjouei | 67 (median) 57-76 IQR | M 10549 (51%) F 9949 (49%) | 27.8 (median) 24.3-32 IQR | I: 367 (1.8%), II: 8290 (40%), III: 10955 (53%), IV: 866 (4.3%) | Diabetes - 14% NIDDM, 5.6% IDDM. Smoker - 12%. Dyspnoea - 0.3% at rest, 8.1% at moderate exertion. COPD - 4.6%. CHF 0.9%. | Non metastatic |
| Farah | Median/mean not reported for entire cohort | Median/mean not reported for entire cohort | Median/mean not reported for entire cohort | - | Median/mean not reported for entire cohort | Non metastatic |
| Maeda | 80.5 median (75-95) TO, 80 median (75-92) non TO | M 103 (55.3%) F83 (47.7%) | 22.4 (TO), 22.4 (non-TO) | I/II/III - 0/45/35% vs 2/48/56% p=0/16 TO vs nTO | Hypertension 42.5% TO 40.5% Non TO. Diabetes 13.7% TO 16% non TO. CVD 7.5% TO 11.3% non TO. Pulmonary disease 3.7% TO 11.3% non TO | I/II/III - TO vs non TO - 23/33/24% vs 38/41/27% |
| Rubio Garcia | 68.8 (mean) +/- 11 | M 337 (59.6%), F 227 (40.4%) | - | I-II: 320 (56.7%), III-IV: 244 (43.6%) | - | T0 10.8%, T1 7.3%, T2 14.7%, T3 50.4%, T4 16.8%, N0 64.2%, N+ 35.8% |
| Manatakis | 70.7 mean +/- 11.4 | M 78 (60.9%), F 50 (39.1%) | - | II: 92 (71.9%), III: 36 (28.1%) | - | pT1 22 (17.2%), pT2 16 (12.5%), pT3 73 (57%), pT4 17 (13.3%). pN0 84 (65.6%), pN (+) 44 (34.4%) |
| Shaikh | 76 median (70-82 IQR) | M 18906 (46.2%) F 22033 (53.8%) | - | - | - | Early T stage vs advanced T stage OR 0.82 for achieving TO CI 0.78-0.86 (p<0.001) |
| Tetley | 55.2 mean +/- 13.4 | M 572 (52.1%) F 506 (47.9%) | - | - | - | - |
| Taffurelli | 80 median (range 70-92) | M 223 (53%) F 198 (47%) | Median 26 (range 16-43) | I-II: 152 (36.1%), III-IV: 269 (63.9%) | Age-adjusted Charlson Age Comorbidity Index score ≤ 6 211 (50.1%), >6 210 (49.9%) | T0 54 (12.8%), T1 94 (22.3%), T2 142 (33.7%), T3 109 (25.4%), T4 24 (5.7%) |
| Naffouje | 62 median (range 54-71) | M 5550 (62.6%) F 3319 (37.4%) | - | - | - | T1 62 (0.7%), T2 397 (4.5%), T3 7663 (86.4%), T4 747 (8.4%); N0 4135 (46.6%), N1 3951 (4.5%), N2 783 (8.8%), Stage II 4135 (46.6%), Stage III 4734 (53.4%); Well differentiated 627 (7.1%), moderately differentiated 6084 (68.6%), poorly differentiated 815 (9.2%), not reported 1343 (15.1%) |
| Yang | 65.2 mean, 67 median (21-96 range) | M 465 (57.9%) F 339 (42.1%) | - | - | - | I/II - TO vs non-TO - 226 vs 134, III/IV - TO vs non-TO - 252 vs 192, T 1-2 - TO vs non-TO - 86 vs 53, T3-4 392 vs 273, N0 - TO vs non-TO - 236 vs 139, N1-2 - TO vs non-TO - 242 vs 187 |
| van Groningen | ≤60 years: 1726, 61-70 years: 4847, 71-80 years: 3288, ≥81 years: 1510 | M 6473 (56.9%) F 4900 (43.1%) | <18.5 - colon vs rectum - 130 (1.6%) vs 46 (1.4%), 18.5-25: 3019 (36.9%) vs 1216 (38.1%), 25-30: 3161 (38.6%) vs 1216 (38.1%), 30+: 1578 (19.3%) vs 538 (16.8%), unknown: 293 (3.6%) vs 70 (2.2%) | I-II - colon vs rectum 6161 (75.3%) vs 2680 (83.9%) , III - colon vs rectum - 1882 (23.0%) vs 494 (15.5%) , IV-V - colon vs rectum - 136 (1.7%) vs 20 (0.6%) | - | pT0/1 - colon vs rectum - 1043 (12.8%) vs 633 (19.9%), pT2 - colon vs rectum - 1399 (17.1%) vs 985 (30.9%), pT3 - colon vs rectum - 4325 (53.0%) vs 1439 (45.1%), pT4 - colon vs rectum - 1381 (16.9%) vs 125 (3.9%), missing - colon vs rectum - 10 (0.1%) vs 6 (0.2%) |
| Mehta | 73 median (IQR 69-78) | Colorectal specific not reported | - | - | - | - |
| Kolfschoten | >70 years: 2403 (45%), 70-79 years: 1769 (33%), >80 years: 1212 (22%) | M 3020 (48%) F 2562 (52%) | - | III+ 1388 (26%) | - | I 949 (18%), II 1932 (36%), III 819 (15%), IV 819 (15%), X 65 (1%) |
| Paro | 75 median (IQR 70–80) | Colorectal specific not reported | - | - | - | - |
| Warps | >75 years 5324 (25.9%) | M 12884 (62.8%) F 7637 (37.2%) | <18.5 303 (1.5%), 18.5-30 16340 (79.6%), >=30 3531 (17.2%) | III+ 3785 (18.4%) | - | T1-2 5688 (27.7%), T3 12036 (58.7%), T4 2033 (9.9%). N0 8892 (43.3%), N1-2 10949 (53.4%) |

Table S3 Study textbook outcome definition and proportion of patients achieving textbook outcome and elective status

| Author | Textbook outcome definition | Textbook outcome percentage | Elective or urgent? |
| --- | --- | --- | --- |
| Ganjouei | No postoperative complications, reinterventions, readmissions, mortality within 30 days of surgical procedure, and hospital LOS (length of stay) of ≤5 days (75th percentile in cohort). Post operative complications = anastomotic leak, ileus (>3 days of NPO or NGT use), superficial and deep SSI, intraabdominal abscess, wound dehiscence, DVT, PE, acute kidney and renal failure, UTI, stroke, cardiac arrest, MI, pneumonia, reintubation, failure to wean ventilator, bleeding, sepsis, septic shock, C.diff infection | 13529/20498 (66%) | Elective |
| Farah | LOS <5 days (75th percentile in cohort), absence of 30-day complications, 30-day readmission, 30-day mortality. Major morbidity = complication adapted Clavien-Dindo (CD) classification to ACS-NSQIP. | Right hemicolectomy 4684/7056 (66.4%) after 2:1 propensity matching, Left hemicolectomy 6585/9369 (70.3%) after 2:1 propensity matching, Low anterior resection 6557/9708 (67.5%) Total using TO and propensity matched cohort group = 17826/26133 (68.2%) | Elective |
| Maeda | Receiving optimal oncologic therapy in treatment of non-metastatic invasive CRC. 5 measures - surgery within 6 weeks, radical resection, LN yield ≥12, no stoma, no adverse outcome (using CD Classification system or readmission <30 days) | 80/186 (43%) | Elective |
| Rubio Garcia | Radical resection (R0) with surgical margins without macro/micro tumour involvement, LN isolated (figure <12 adequate for staging, in accordance with AJCC guidelines), No complications >CD III during first 30 days, hospital stay <14 days <75th percentile, no readmission/mortality first 30 days | 281 (49.8%) | Elective |
| Manatakis | Achieving all 6 outcomes - hospital survival, radical resection (R0), no major complication (> CD II), no reintervention, no unplanned stoma, no prolonged LOS (>75th percentile at 11 days) or 30-day readmission | 77 (60.2%) | 92.2% elective 7.8% urgent |
| Shaikh | Absence of any postoperative complications (not defined), prolonged length of stay(>75th percentile in cohort), 90-day mortality, readmission within 90 days of discharge | 23580 (57.6%) | 27590 (67.5%) elective 13290 (32.5%) urgent |
| Tetley | Surgeries with the absence of 30-day CD IV complications, unplanned reoperations, 30-day mortality after the date of surgery, 30-day readmissions after the date of discharge from the index hospitalization, and emergency department or observations stays (EDOS) | Overall 669 (62.9%) [Private 195 (77.4%), Medicare 123 (59.4%), Medicaid/ uninsured 351 (56.7%)] | 620 (57.5%) elective, 340 (31.5%) urgent, 118 (10.9%) emergent |
| Taffurelli | 90-day survival after surgery, No 90-day postoperative complications CD>3, no need for reintervention, no readmission, no need for discharge to rehabilitation/ nursing home facilities, no post operative changes in living situations, and length of stay ≤ 5 days for colon cancer surgery or ≤ 14 days for rectal cancer surgery | 288 (68.4%) | Elective |
| Naffouje | Achievement of negative distal and circumferential resection margin (CRM), retrieval of ≥ 12 nodes, no 90-day mortality, and length of stay (LOS) < 75th percentile of corresponding year’s range. | 3967 (44.7%) | - |
| Yang | Patients who had colon cancer surgery and surgery within 6 weeks, radical resection, lymph node (LN) yield ≥12, no ostomy, no adverse outcome (any adverse outcome occurring, including readmission or reoperation within 30 days after resection) and colonoscopy before/after surgery within 6 months | 478 (59.5%) | - |
| van Groningen | Colon TO = Hospital and 30-day survival, negative resection margins, no need for reintervention, no ostomy, no adverse outcome and a hospital stay of <14 days. Rectum TO = Circumferential resection margin involvement. Both TO = Postoperative complication [Any adverse event within 30 days after surgery (including everything from a urinary tract infection to anastomotic leakage or death)], Serious complication [Patients with a postoperative complication after surgery leading to an in-hospital stay of more than 14 days, a surgical, endoscopic or radiological reintervention, or to death]; 30 day mortality [Patients that died within 30 days after surgery or during the first hospital admission.]; Failure to rescue (FTR) [Patients that died within 30 days after surgery or during the first hospital admission after a serious complication] | 5113 (62.9%) | Colon 2015: 86.4% elective, 13.6% nonelective Rectum 2015: 99.1 elective, 0.9% nonelective Colorectal 2013-2015: 88.1% elective11.9% nonelective |
| Mehta | No postoperative surgical complications (ICD-9-CM diagnosis and procedure codes were utilized to identify patients having postoperative surgical complications), no prolonged length of hospital stay, no readmission 90 days after discharge, and no postoperative mortality 90 days after surgery | 5440 (49.5%) | Elective |
| Kolfschoten | Post-operative mortality (mortality within the hospitalization or 30 days after resection), radical resection, no reintervention (an adverse outcome requiring a reoperation or percutaneous reintervention), no ostomy placement, no adverse outcomes (any adverse outcome occurring within 30 days after resection) and a hospital stay of 14 days or less. | 2721 (49%) | 19% urgent |
| Paro | No 90-day mortality, no 90-day readmission, no postoperative surgical complications (identified using ICD-9-CM and ICD-10-CM codes and included pulmonary failure, pneumonia, myocardial infarction, deep venous thrombosis, pulmonary embolism, renal failure, surgical site infection, gastrointestinal bleeding, and postoperative haemorrhage), and no prolonged length of stay (LOS was defined as LOS during index hospitalization longer than the 75th percentile) | Overall 67448 (53.6%) [colon 60151 (54.1%), rectal 7297 (49.6%)] | 111,185, 55.5%) were non-urgent (n = 156,059, 77.8%) |
| Warps | 30 day primary hospital admission survival, no reintervention, tumour free margins, no postoperative complications, LOS <14 days, no readmission | 11556 (56.3%) | Elective |

Table S4 Perioperative and post operative outcomes

| Author | Mortality | Length of stay | Type of surgery | Lymph node yield | Reoperation | Complication | No stomy | Radical resection |
| --- | --- | --- | --- | --- | --- | --- | --- | --- |
| Ganjouei | 30 day mortality 0.7% | ≤5 days 76%, >5 days 24%. | Textbook outcome was more frequently after robotic colectomy (77%), followed by lap colectomy (68%), then open colectomy (39%), p<0.001 | - | Reoperation within 30 days - 2.7% | - | - | - |
| Farah | Left anterior resection - 20/4854 vs 18/4854 30 day mortality 0.4% vs 0.4% p=0.871 (Robotic vs laparoscopic). Right Colectomy - 45/4704 vs 16/2352 30 day mortality 0.7 vs 1% p=0.17 (robotic vs laparoscopic). Left colectomy - 48/6246 vs 29/3123 30 day mortality 0.9 vs 0.8% p=0.38 (robotic vs laparoscopic) | Right colectomy 4.7 vs 4 p<0.001 (laparoscopic vs robotic), Left colectomy 4.6 vs 4 p<0.001 (laparoscopic vs robotic), Left anterior resection 4.8 vs 4.7 p<0.001 (laparoscopic vs robotic) | Right colectomy - laparoscopic 4704, robotic 2352. Left colectomy - laparoscopic 6246, robotic 3123. Left anterior resection- laparoscopic 4854, robotic 4854 | - | - | Right colectomy laparoscopic vs robotic 779/4704 (16.6%) vs 375/2352 (15.9%); Left colectomy laparoscopic vs robotic 870/6246 (13.9%) vs 398/3123 (12.7%); Left anterior resection laparoscopic vs robotic 580/4854 (11.9%) vs 591/4854 (12.2%) | - | - |
| Maeda | - | - | - | D1/D2/D3 - TO vs non-TO - 0/20/60 vs 2/45/59 p=0.01 | - | 140/186 75.2% (no adverse outcome) | 87% | 97.30% |
| Rubio Garcia | 30 day mortality 4.3% | 78.6% hospital stay <14 days | Right hemicolectomy 221 (39.1%), sigmoidectomy 210 (37.2%), left hemicolectomy 62 (11%) | Number of isolated lymph nodes 18.3 mean (8.7 SD) | - | 24.6% (intraabdominal collection 21 patients 3.71%, evisceration 20 patients 3.54%, anastomotic leak 46 patients 8.15%) | 90.80% | - |
| Manatakis | 30 day mortality 4 (3.1%) | 9.9 mean days (7.7 days SD) | R0 124 (96.9%), R1 4 (3.1%) | ≥12 LN 122 (95.3%). Mean lymph node yield 25.5 +/-11.4 (range 6-74) | 10.20% | 69.60% | 97.70% | 92.20% |
| Shaikh | 90 day mortality 2402 (5.9%) | Prolonged LOS 7433 (18.2%) | - | - | - | 8813 (21.5%) | - | - |
| Tetley | 30 day mortality overall 26 (2.4%), private 3 (1.2%), Medicare 8 (3.9%), Medicaid/ Insurance 15 (2.4%) | LOS mean (SD): Overall 10.5 (11.5), Private 8.7 (11.2), Medicare 11.0 (10.5), Medicaid/ Uninsured 11.1 (11.8) | Laparoscopic 431 (40%) [Private 116 (46%), Medicare 90 (43.5%), Medicaid/ Uninsured 225 (36.3%)], Open abdomen 647 (60%) [Private 136 (54%), Medicare 117 (56.5%), Medicaid/ Uninsured 394 (63.7%) | - | 88 (8.2%) [Private 16 (6.3%), Medicare 20 (9.7%), Medicaid/ Uninsured 52 (8.4%)] | - | - | - |
| Taffurelli | 90 day mortality 12 (2.9%) | 87 (20.7%) prolonged LOS (≤5 days for rectal cancer, ≤14 days for rectal cancer surgery) | Laparoscopic 412 (97.7%), Open 9 (2.3%), converted to open 4 (1%). Procedure : right colectomy 210 (47.7%), left colectomy 73 (17.3%), subtotal colectomy 31 (7.4%), low anterior resection (partial mesorectal excision) 28 (6.7%), low anterior resection (total mesorectal excision) [abdominoperineal resection] 88 (55%) [20.9%]. Anastomosis: Primary anastomosis without stoma 308 (73.2), anastomosis with loop ileostomy 38(9%), end colostomy 75 (17.8%). Extended resection: No 401 (95.2%), Yes 20 (4.8%) | - | 19 (4.5%) | - | - | - |
| Naffouje | 90 day mortality 173 (2.0%) | LOS <75th percentile: 1876 (21.2%) | Low anterior resection 6037 (68.1%), abdominoperineal resection 2591 (29.2%), exenteration 241 (2.7%), open 5006 (56.4%), laparoscopic 2207 (24.9%), robotic 1656 (18.7%) | <12: 2612 (29.5%), >12: 6257 (70.5%) | - | - | - | - |
| Yang | - | - | - | 714 (88.8%) | - | 121 (15%) | 702 (87.3%) | 756 (94%) |
| van Groningen | 30 day mortality Colon 188 (2.3%), rectum 39 (1.2%), colorectal (total) 227 (2.0%) | - | - | - | - | Postoperative complications: colon 2274 (27.8%), rectum 1186 (37.1%); serious complications: colon 1227 (15%), rectum 635 (19.8%) | | - |
| Mehta | 90 day mortality 404 (3.7%) | No prolonged LOS 8496 (77.3%) | - | - | - | 2780 (25.3%) | - | - |
| Kolfschoten | 270 (5%) | 4319 (77%) LOS <14 days | - | - | 866 (13%) | 1851 (33%) | 835 (15%) | 4965 (89%) |
| Paro | 90 day mortality colon 9.5%, rectal 6.2% | 22.8% colon, 22.8% rectal | - | - | - | Colon 26.2%, rectal 21.0% | - | - |
| Warps | 30 day mortality 20303 (98.9%) | 17316 (84.4%) | Open 3828 (18.7%), laparoscopic 16501 (80.4%), missing 192 | - | 2759 (13.4%) | No complications 7396 (36%) | 5977 (29.1%) rectum resection with definitive stoma | - |

Table S5 Readmission rates and textbook outcome predictive factors

| Author | Readmission | Predictive factors |
| --- | --- | --- |
| Ganjouei | 30 day readmission - 6.4% | Predictive pre-operative variables, TO vs non-TO - surgical approach (TO was more frequently achieved after robotic colectomy (77%), followed by laparoscopic colectomy (68%), then open colectomy (39%), p<0.001), patient age (65yo vs 71 yo p<0.001), pre-op haematocrit (39.1% vs 36.6% p<0.001), pre-op oral antibiotics, bowel prep (68% vs 57% p<0.001), female sex (50% vs 46% p<0.001). |
| Farah | 30 day readmission- Left anterior resection 9.1 vs 10.4% p=0.023 (laparoscopic vs robotic), Right colectomy 8.4% 396/4704 vs 8.4% 197/2352 p=0.97 (laparoscopic vs robotic), Left colectomy 7% 435/6246 vs 7.1% 223/3123 p=0.729 (laparoscopic vs robotic) | - |
| Maeda | - | Kaplan Meier analysis - TO significant predictor for 5 year OS and RFS compared to non-TO - (OS, 77.8% vs. 60.8%, P < 0.01; RFS, 69.6% vs. 50.8%, P = 0.01). Multivariate analysis - non-TO was an independent predictive factor for worse OS (HR, 2.04; 95% confidence interval (CI), 1.175–3.557; P = 0.01) and RFS (HR, 1.72; 95% CI, 1.043–2.842; P = 0.03). |
| Rubio Garcia | 30 day readmission 11.7% | T3 and T4 classification (OR 2.50, 95% CI 4.59–1.36, and OR 2.55, 95% CI 5.21–1.24 respectively) and laparoscopic approach (OR 1.53, 95% CI 2.33–1.00) were independent factors that were significantly associated with achieving a TO |
| Manatakis | 30 day readmission 4 (3.1%) | Older age, left-sided resections and pT4 tumours limit TO (P=0.005, p =0.013, p= 0.012 respectively). The 5-year overall and 5-year cancer-specific survival significantly better in the TO versus non-TO subgroup (81% vs. 59%, p = 0.009, and 86% vs. 65%, p = 0.02, respectively). |
| Shaikh | 90 day readmission 9105 (22.2%) | Multivariable analysis: Patients in high EQI (environmental quality index) areas less likely to achieve TO (OR: 0.94, 95% confidence interval 95% CI 0.89–0.99; p = 0.02). Black patients living in moderate-to-high EQI countries 30% less likely to reach TO compared to white patients in low EQI counties ( OR 0.69 95% CI 0.55-0.87) |
| Tetley | 30 day readmission: Overall 223 (20.7%) [Private 37 (14.7%), Medicare 40 (19.3%), Medicaid/ Uninsured 146 (23.6%)] | Private patients were more likely to achieve TO when compared to Medicare and Medicaid/Uninsured patients (79.4% vs 67.4% vs 62.0% for elective cases and 72.7%, 46.2%, and 51.2% for urgent/emergent cases respectively) |
| Taffurelli | 30 day readmission 48 (11.4%) | Risk factors preventing TO on univariate analysis: dependent in living situation (p = 0.041), an ASA score ≥3 (p = 0.012), a CACI (Charlson age comorbidity index) >6 (p = 0.001), an fTRST (Flemish version of the Triage Risk Screening Tool) ≥2 (p < 0.001), ADL (Activities of Daily Living) <5 (p = 0.030), a TUG (Timed Up & Go) >20 s (p = 0.001), ECOG-PS (Eastern Cooperative Oncology Group – Performance status) >1 (p = 0.011) and ileostomy creation (p = 0.045). Factors increasing risk of unfavourable post operative outcomes on multivariate analysis, patients with an fTRST ≥2 (OR 1.97, 95% CI 1.23–3.16; p = 0.005),a CACI >6 (OR 1.61, 95% CI 1.03–2.51; p = 0.036) or a TUG >20sec (OR 2.06, 95% CI 1.01–4.19; p = 0.048). |
| Naffouje | 30 day readmission 625 (7.0%) | Factors reducing odds of attainment of TO on multivariable analysis: increasing age (HR 0.992 95% CI 0.988-0.996 p<0.001), male gender (female gender HR 1.117 95% CI 1.023-1.220 p=0.014), Black race (HR 0.741 95% CI 0.628-0.875 p<0.001), higher Charlson score (for Charlson score 2, 3+ respectively: HR 0.723 95% CI 0.582-0.898 p=0.003, HR 0.702 95%CI 0.514-0.958 p=0.026), public insurance (Private insurance HR 1.307 95% CI 1.179-1.450 p<0.001), cT4 lesions (HR 0.581 95%CI 0.455-0.741 p<0.001), undergoing abdominoperineal resection or exenteration as opposed to left anterior resection (APR: HR 0.752 95%CI0.66-0.799 p<0.001, Exenteration: HR 0.692 95% CI 0.521-0.919 p<0.001), open approach (minimally invasive surgery HR 1.137 95% CI 1.039-1.243 p=0.005) and care at low-volume centres (high volume centre HR 1.205 95% CI 1.102-1.318 p<0.001). |
| Yang | - | - |
| van Groningen | - | - |
| Mehta | 90 day readmission 2892 (26.3%) | - |
| Kolfschoten | - | Factors which influence TO. Age (with age 70 as reference): Age 70-79, HR 0.66 95% CI 0.57-0.75, Age 80+ HR 0.49 95% CI 0.41-0.57. Female HR 1.51 95% CI 1.34-1.71. Abdominal history HR 0.82 95%CI 0.72-0.93. ASA III+ HR 0.52 95% CI 0.46-0.6. Acute case HR 0.44 95% CI 0.35-0.56. Perforation HR 0.4 95% CI 0.23-0.69. Obstruction HR 0.66 95%CI 0.51- 0.86. Left hemicolectomy HR 0.77 95% CI 0.46-0.92. Left anterior resection HR 0.46 95% CI 0.38-0.55. TNM Stage II HR 0.74 95%CI 0.62-0.88, Stage III HR 0.74 95% CI 0.62-0.88, TNM Stage IV HR 0.21 95% CI 0.16-0.25. Local invasion HR 0.44 95% CI 0.36-0.55. |
| Paro | 90 day readmission: colon 17%; rectal 27.5% | Predictive factors in attainment of TO: Teaching hospital status (OR 0.97, 95% CI 0.96–0.99 p < 0.05). Patients living in high racial integration areas vs low racial integration areas/segregated counties (55.0% vs. 59.3%; p < 0.001). |
| Warps | 30 day readmission: No readmissions 17662 (85.9%) | Factors negatively associated with TO: age >= 75 years (Adjusted Odds Ratio (AOR) 0.912, 95% CI 0.846-0.984), BMI >=30 kg/m2 (AOR 0.747, 95% CI 0.689-0.810), ASA-score III+ (AOR 0.676, 95% CI 0.621-0.736), CCI II+ (AOR 0.809, 95% CI 0.749-0.873), preoperative tumour-related complications (AOR 0.893, 95% CI 0.822-0.971), neoadjuvant (chemo)radiotherapy (Short course radiotherapy: AOR 0.761, 95% CI 0.695-0.834, Chemoradiotherapy: AOR 0.888, 95% CI 0.801-0.984), other radiotherapy scheme: AOR 0.724, 95% CI 0.555-0.944), multivisceral resection for local ingrowth (AOR 0.617, 95% CI 0.533-0.715), (y)pT3 and (y)pT4 stage (AOR 0.787, 95% CI 0.718-0.863 and AOR 0.575 95% CI 0.478-0.692 respectively), Surgery in a tertiary hospital (AOR 0.775, 95% CI 0.646-0.929). Factors positively associated with TO: female gender (AOR 1.599, 95% CI 1.499-1.706), laparoscopic surgery (AOR 1.323, 95% CI 1.201-1.458), type of procedure - rectal resection without defunctioning stoma (AOR 1.934, 95% CI 1.773-2.109), low Hartmann's procedure (AOR 1.328, 95% CI 1.196e1.474) and Abdominoperineal resection (AOR 1.105, 95% CI 1.021e1.208). |
